# Supplementary material for: Prevalence of dural puncture headache after caesarean section at a tertiary hospital in the Gambia
Source: PLoS One. 2025 Sep 19;20(9):e0303410. doi: 10.1371/journal.pone.0303410 (PMC12448977; doi:10.1371/journal.pone.0303410)
Supplement: Attachment — Submitted filename: PONE-D-24-11503.pdf [file pone.0303410.s002.pdf]

PLOS ONE

Prevalence of Dural Puncture headache after caesarean section at a Tertiary Hospital in the Gambia.

--Manuscript Draft--

**Manuscript Number:**	PONE-D-24-11503
**Article Type:**	Research Article
**Full Title:**	Prevalence of Dural Puncture headache after caesarean section at a Tertiary Hospital in the Gambia.
**Short Title:**	Prevalence of Dural Puncture headache in Gambia
**Corresponding Author:**	Matthew Oguwike Anyanwu, M.D, FWACS<br>Edward Francis Small Teaching Hospital<br>Banjul, GAMBIA
**Keywords:**	Keywords: Post-Dural Puncture Headache (PDPH), Caesarean Section, Prevalence, Spinal
**Abstract:**	Background:<br>Following a caesarean section performed under spinal anaesthesia, a common complication is post-dural puncture headache (PDPH). Spinal anaesthesia has become the most common anaesthetic procedure during caesarean section in our practice. Therefore, knowing the prevalence and risk factors of PDPH will inform practice and add value in our obstetrics practice.<br>Methodology:<br>A cross-sectional study was conducted at Edward Francis Small Teaching Hospital Banjul and data was effectively collected from August to October 2023. A structured data collection tool was used. The data was entered into a computer database and analyzed using the SPSS version 26. Pain was graded using a 10-cm visual analogue scale. Results were expressed in simple descriptive statistics and test of significance was set at p-value 0.05.<br>Results:<br>The overall prevalence of Post Dural Puncture Headache (PDPH) was 42.7% with majority of cases presenting with Occipital headache (29.2%), lasting for >3hrs (18%). A statistically significant association between PDPH and Gestational age with p-value (p=0.02); number of attempts with p-value (p=0.01); larger needle gauge and number of CSF drops (p=0.01), respectively was observed.<br>Conclusion:<br>Prevalence of PDPH was high and associated with the use of large needle gauges, multiple attempts and increased CSF drops. Patients that underwent emergency CS, have a higher risk of developing Post-Dural Puncture Headache.
**Order of Authors:**	Matthew Oguwike Anyanwu, M.D, FWACS
	Admire Coker, MBChB
**Additional Information:**	
**Question**	**Response**
**Financial Disclosure**<br><br>Enter a financial disclosure statement that describes the sources of funding for the work included in this submission. Review the submission guidelines for detailed requirements. View published research articles from *PLOS ONE* for specific examples.	The author(s) received no specific funding for this work.

Powered by Editorial Manager® and ProduXion Manager® from Aries Systems Corporation

This statement is required for submission and **will appear in the published article** if the submission is accepted. Please make sure it is accurate.<br><br>**Funded studies**<br>Enter a statement with the following details:<br>• Initials of the authors who received each award<br>• Grant numbers awarded to each author<br>• The full name of each funder<br>• URL of each funder website<br>• Did the sponsors or funders play any role in the study design, data collection and analysis, decision to publish, or preparation of the manuscript?<br><br>Did you receive funding for this work?	
**Competing Interests**<br><br>Use the instructions below to enter a competing interest statement for this submission. On behalf of all authors, disclose any competing interests that could be perceived to bias this work—acknowledging all financial support and any other relevant financial or non-financial competing interests.<br><br>This statement is required for submission and **will appear in the published article** if the submission is accepted. Please make sure it is accurate and that any funding sources listed in your Funding Information later in the submission form are also declared in your Financial Disclosure statement.<br><br>View published research articles from *PLOS ONE* for specific examples.	The authors have declared that no competing interests exist.

**NO authors have competing interests**<br><br>Enter: *The authors have declared that no competing interests exist.*<br><br>**Authors with competing interests**<br><br>Enter competing interest details beginning with this statement:<br><br>*I have read the journal's policy and the authors of this manuscript have the following competing interests: [insert competing interests here]*<br><br>* typeset	
**Ethics Statement**<br><br>Enter an ethics statement for this submission. This statement is required if the study involved:<br><br>• Human participants<br>• Human specimens or tissue<br>• Vertebrate animals or cephalopods<br>• Vertebrate embryos or tissues<br>• Field research<br><br>Write "N/A" if the submission does not require an ethics statement.<br><br>General guidance is provided below. Consult the submission guidelines for detailed instructions. **Make sure that all information entered here is included in the Methods section of the manuscript.**	Ethical approval was requested and secured from Research and Ethics Committee of Edward Francis Small Teaching Hospital. The letter of approval was used to have access to patients and medical files at the Obstetrics and Gynaecology department. Informed consent was obtained from all participants and personal information were confidential. Consent for publication was not applicable as there was no minor in the subjects. The approval letter for the study can be uploaded on your request. The consent form used for the study can be uploaded on your request.

Powered by Editorial Manager® and ProduXion Manager® from Aries Systems Corporation

**Format for specific study types**<br><br>**Human Subject Research (involving human participants and/or tissue)**<br>• Give the name of the institutional review board or ethics committee that approved the study<br>• Include the approval number and/or a statement indicating approval of this research<br>• Indicate the form of consent obtained (written/oral) or the reason that consent was not obtained (e.g. the data were analyzed anonymously)<br><br>**Animal Research (involving vertebrate animals, embryos or tissues)**<br>• Provide the name of the Institutional Animal Care and Use Committee (IACUC) or other relevant ethics board that reviewed the study protocol, and indicate whether they approved this research or granted a formal waiver of ethical approval<br>• Include an approval number if one was obtained<br>• If the study involved *non-human primates*, add *additional details* about animal welfare and steps taken to ameliorate suffering<br>• If anesthesia, euthanasia, or any kind of animal sacrifice is part of the study, include briefly which substances and/or methods were applied<br><br>**Field Research**<br><br>Include the following details if this study involves the collection of plant, animal, or other materials from a natural setting:<br>• Field permit number<br>• Name of the institution or relevant body that granted permission	
**Data Availability**<br><br>Authors are required to make all data underlying the findings described fully available, without restriction, and from the time of publication. PLOS allows rare exceptions to address legal and ethical concerns. See the PLOS Data Policy and FAQ for detailed information.	Yes - all data are fully available without restriction

A Data Availability Statement describing where the data can be found is required at submission. Your answers to this question constitute the Data Availability Statement and **will be published in the article**, if accepted.<br><br>**Important:** Stating 'data available on request from the author' is not sufficient. If your data are only available upon request, select 'No' for the first question and explain your exceptional situation in the text box.<br><br>Do the authors confirm that all data underlying the findings described in their manuscript are fully available without restriction?	
**Describe where the data may be found in full sentences. If you are copying our sample text, replace any instances of XXX with the appropriate details.**<br><br>• If the data are **held or will be held in a public repository**, include URLs, accession numbers or DOIs. If this information will only be available after acceptance, indicate this by ticking the box below. For example: *All XXX files are available from the XXX database (accession number(s) XXX, XXX.*).<br>• If the data are all contained **within the manuscript and/or Supporting Information files**, enter the following: *All relevant data are within the manuscript and its Supporting Information files.*<br>• If neither of these applies but you are able to provide **details of access elsewhere**, with or without limitations, please do so. For example:<br><br>*Data cannot be shared publicly because of [XXX]. Data are available from the XXX Institutional Data Access / Ethics Committee (contact via XXX) for researchers who meet the criteria for access to confidential data.*<br><br>*The data underlying the results presented in the study are available from (include the name of the third party*	N/A

and contact information or URL).<br>• This text is appropriate if the data are owned by a third party and authors do not have permission to share the data.<br><br>* typeset	
Additional data availability information:	Tick here if your circumstances are not covered by the questions above and you need the journal's help to make your data available.; Tick here if the URLs/accession numbers/DOIs will be available only after acceptance of the manuscript for publication so that we can ensure their inclusion before publication.

Powered by Editorial Manager® and ProduXion Manager® from Aries Systems Corporation

Manuscript

Click here to access/download;Manuscript;manuscript of postdural puncture head study.docx

Prevalence of Dural Puncture headache after caesarean section at a Tertiary Hospital in the Gambia.

Authors: Matthew Anyanwu[1, 2], Admire Coker[1, 2], Donkor Simon[3]

Affiliation: 1. Edward Francis Small Teaching Hospital
Banjul; The Gambia

2. School of Medicine and Allied Health Sciences University of The Gambia
Kanifing; The Gambia

3. Medical Research Laboratories
Fajara; The Gambia.

Corresponding Author: Matthew Anyanwu. Email: anyanwum@yahoo.com, manyanwu@utg.edu.gm

Abstract

Background:

Following a caesarean section performed under spinal anaesthesia, a common complication is post-dural puncture headache (PDPH). Spinal anaesthesia has become the most common anaesthetic procedure during caesarean section in our practice. Therefore, knowing the prevalence and risk factors of PDPH will inform practice and add value in our obstetrics practice.

Methodology:

A cross-sectional study was conducted at Edward Francis Small Teaching Hospital Banjul and data was effectively collected from August to October 2023. A structured data collection tool was used. The data was entered into a computer database and analyzed using the SPSS version 26. Pain was graded using a 10-cm visual analogue scale. Results were expressed in simple descriptive statistics and test of significance was set at p-value 0.05.

Results:

A total of 89 participants with mean age of 28 years (SD±6.1) and majority 45(50.6%), between 21 to 30 years. Majority had low parity (0 to 3), 64(71.9%), emergency CS of 71(79.8%), while only 20.2% (n=18) were elective CS. Overall prevalence of Post Dural Puncture Headache (PDPH) was 42.7% with majority of cases presenting with Occipital headache (29.2%), lasting for 3hrs (18%). A statistically significant association between PDPH and Gestational age with *p-value (p=0.02)*; number of attempts with *p-value (p=0.01);* larger needle gauge and number of CSF drops (p=0.01), respectively were observed.

Conclusion:

Prevalence of PDPH was high and associated with the use of large needle gauges, multiple attempts and increased CSF drops. Patients that underwent emergency CS, have a higher risk of developing Post-Dural Puncture Headache.

Key[illegible]rds: Post-Dural Puncture Headache (PDPH), Caesarean Section, Prevalence, Spinal

Introduction

Spinal anaesthesia is a neuraxial anaesthesia technique in which local anaesthetic is placed directly in the intrathecal space (subarachnoid space). The subarachnoid space houses sterile cerebrospinal fluid (CSF), the clear fluid that bathes the brain and spinal cord. There are roughly about 130 to 140 mL of CSF in an adult human which continually cycles throughout the day (1).

Subarachnoid (spinal) block is a safe and effective alternative to general anaesthesia when the surgical site is located on the lower extremities, perineum (e.g. surgery on the genitalia or anus), or lower body wall (e.g. inguinal herniorrhaphy) (2). Subarachnoid blocks (SAB) popularly known as spinal anaesthesia however, is cheaper and brings maternal satisfaction to many mothers but is not devoid of some complications, among which is post-Dural puncture headache (PDPH) (3).

Post-Dural puncture headache (PDPH) is a headache that may occur 24 to 48 hours after spinal anaesthesia (or inadvertent epidural subarachnoid puncture). It is thought to be due to a continuous leak of CSF through the spinal puncture hole, decompressing the subarachnoid space with secondary stretching of the cranial nerves (4). The pathophysiology of spinal headache is thought to be related to spinal fluid leakage resulting in traction on the meninges and blood vessels and subsequent vasodilation of the cerebral vasculature (5). This leads to low CSF pressure following leakage of CSF through the dural puncture hole and the choroid plexus is unable to secrete sufficient fluid to maintain the CSF pressure. This results to Venous dilation and compensatory increase in brain volume will result in brain sag which in turn will exert traction and stimulate pain sensitive anchoring structures like dural vessels, basal dura and tentorium cerebelli, causing post spinal headache. This condition is found to be more frequent in young females and is said to be associated with needle size and type. Symptoms include a throbbing headache related to posture and presents most commonly within 48 hours of CSF hypotension which in turn leads to intracranial venous dilation resulting in an increase in brain volume (3).

Regarding prevalence of PDPH there are many studies in this field more so now that spinal anaesthesia has become a predominant anaesthetic procedure for caesarean section in our sub-region. Some prospective cohort studies conducted in rural India (5) and Northern Nigeria (6)

reported 11.4% and 15.8% incidence of PDPH respectively. However, a retrospective cohort study conducted in Jordan reported a lower incidence of PDPH (6.3%) (7).
A cross sectional study conducted in Ethiopia had incidence of post dural puncture headache 31.3% (8).

Several risk factors have been attributed to PDPH including age, weight, female gender, pregnancy, needle size and design, and number of puncture attempts. Spinal needle size and design has been implicated as risk factors of PDPH (9). Additionally, insertion of the needle with the bevel parallel to the dural fibers facilitates closure of the hole (10). Women for caesarean delivery are at increased risk because of their young age and increased vascular distension response to CSF leakage, due to higher oestrogen level during pregnancy (11). Multiple dural punctures caused by unsuccessful puncture would increase the rate of PDPH (12).

In view of these risk factors that potentially prevails in our practice and the increasing use of spinal anesthesia for caesarean section we decided to conduct the study to show prevalence of Post Dural Puncture Headache and associated symptoms.

Methodology

A cross-sectional study design was used to conduct this research. The study population was women after caesarean section at post-natal ward at Edward Francis Small Teaching Hospital Banjul The Gambia. The proposal was submitted for approval in July and recruitment and data collection effectively started on the 13/08/2023 and ended 05/10/2023. Compilation and analysis was completed on the 16/10/2023.

Sample size was calculated with G power 3.1 software. A sample size of 100 with effect size at 25% = 0.25, Power at 85 , t-te[illegible]t 0.05.

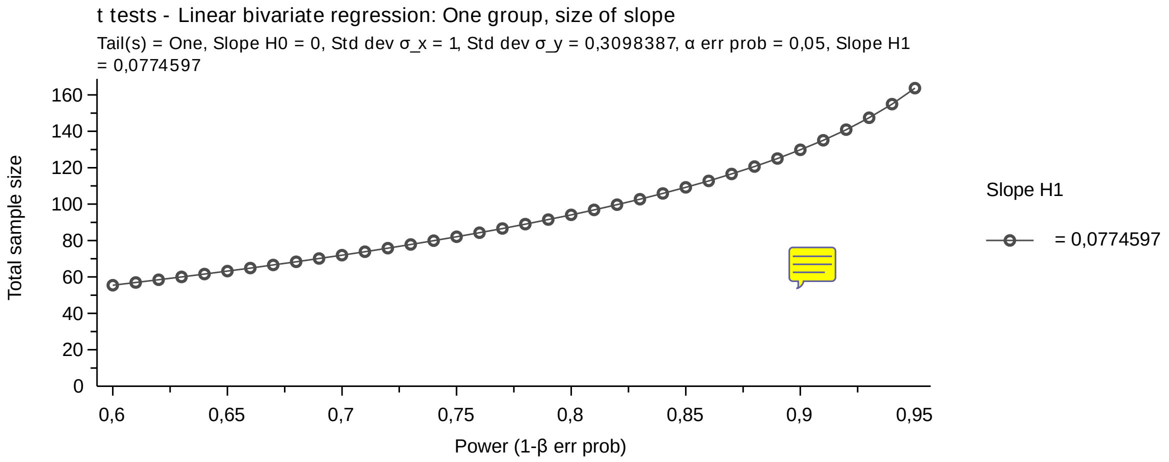

Inclusion and Exclusion criteria

Women who were scheduled for either emergency or elective caesarean section with the ages of 15 – 49yrs. The women who had pre-existing diagnosis of chronic headaches or migraines were excluded as these conditions may complicate the interpretation of PDPH occurrence and symptoms.

Data collection

The study was conducted with modified self-administered structured questionnaire which has both discrete and continuous variables. The biodata, reproductive and social history; spinal anaesthetic characteristics and pregnancy outcomes were the variables in this research. The

outcome measures includes the sociodemographic characteristics, prevalence of PDPH, the associated symptoms and risk factors of PDPH.

Consent form was used and all patients signed the consent form, no minor was in the study.

Data analysis

Data was entered into computer database and consistent checks instituted to ensure errors are corrected before analysis. Data was analyzed with SPSS and EPI Info and results expressed by simple descriptive statistics in tables and graphs. The test of significance p-value at 0.05 was used.

Ethical Considerations

Ethical approval was requested and secured from Research and Ethics Committee of Edward Francis Small Teaching Hospital. The letter of approval was used to have access to patients and medical files at the Obstetrics and Gynaecology department. Informed consent was obtained from all participants and personal information were confidential.

RESULTS

Socio-demographic characteristics

From 93 patients who were recruited for this study, 4 of them were excluded due to some missing information. A total of 89 participants data were included for analysis. Out of 89 patients on the selected sample, majority belong to the age group between 21 to 30 years, 45(50.6%), followed by the age group between 31 to 40 years, 28 (31.5%) and age group between 41 to 50 years, 2(2.2%). The mean age of 28 years (SD±6.1). Majority of patients were married, 79(88.8%), with Muslim religion, 74(83.1%) and a secondary educational level 39(43.8%) predominant.

Table 1. Distribution of women by socio-demographic characteristics. Postnatal ward-EFSTH; August to September 2023. n= 89

Socio-demographic characteristics	N	%
**Age**		
15 to 20	14	15.7
21 to 30	45	50.6
31 to 40	28	31.5
41 to 50	2	2.2
**Marital status**		
Married	79	88.8
Single	10	11.2
**Religion**		
Christian	15	16.9
Muslim	74	83.1
**Level of Education**		
Islam/Arabic	10	11.2
Primary	13	14.6
Secondary	39	43.8

Tertiary	20	22.5
No formal education	7	7.9

Intraoperative data

Among 89 patients who underwent caesarean section under spinal anesthesia, majority of them had a parity of 0 to 3, 64(71.9%), emergency CS of 71(79.8%), while only 20.2% (n=18) were elective CS.

Table 2. Distribution of women by Clinical characteristics (general). Postnatal ward-EFSTH; August to September 2023. n= 89

**Clinical characteristics(general).**	**N**	**%**
**Parity**		
Para (0 to 3)	64	71.9
Para (4 to 8)	25	28.1
**Previous History of CS**		
No	67	75.3
Yes	22	24.7
**Gestational Age (weeks)**		
28 to 31	2	2.2
32 to 36	29	32.6
37 to 41	58	65.2
**Urgency of CS**		
Elective	18	20.2
Emergency	71	79.8
**Duration in Labour (mins)**		
20 to 35	13	14.6

36 to 50	51	57.3
51 to 65	22	24.7
66 to 80	3	3.4

Intraoperative Anesthesia data

In this study, 10mg of Bupivacaine was used in majority 66(74.2%) of the patients and 12.5mg was used in the minority 23(25.8%). A 22G needle was used in 61(68.5%) of patients followed by 25G needle used in 28(31.5%) of patients. [illegible]ttempts were done in the majority of the patients which was 62(69.7%) while only one attempt was done in 27(30.3%) of patients. L3-L4 puncture was done in 74(84.3%) of patients. There were 2 drops 56(62.9%) of cerebrospinal fluid in majority of the patients and 1 drop 33(37.1%).

Table 3. Distribution of women by Clinical characteristics (anaesthesia).

**Clinical characteristics (anaesthesia).**	**N**	**%**
**Dosage of Anaesthetic agent (mg)**		
10	66	74.2
12.5	23	25.8
**Number of attempts**		
1	27	30.3
2	62	69.7
**Size of needle (G)**		
22	61	68.5
25	28	31.5
**Level of Puncture**		
L2-L3	8	9.0
L3-L4	75	84.3

L4-L5	6	6.7
**Number of CSF Drops**		
1	33	37.1
2	56	62.9

Postoperative outcomes

Among 89 women who underwent CS, 38(42.7%) of patients developed PDPH, of which, 16(18%) of the headache lasted for >3hrs. Among 38(42.7%) women who developed PDPH, 17(19.1%) of them suffered severe pain, and 14(15.7%) of them had moderate pain.

Table 4. Distribution of pregnant women by Clinical characteristics (PDPH).

Clinical characteristics (PDPH).	N	%
**Previous history of PDPH**		
No	74	83.1
Yes	15	16.9
**Severity of previous history**		
Mild	4	4.5
Moderate	5	5.6
Severe	74	83.1
Nil	6	6.7
**Post Dural Puncture Headache**		
No	51	57.3
Yes	38	42.7
**Severity of PDPH**		
Mild	7	7.9

Moderate	14	15.7
Severe	51	57.3
Nil	17	19.1
**Duration of Headache**		
>3hrs	16	18.0
1hr	10	11.2
2hrs	12	13.5
Nil	51	57.3
**Location of Headache**		
Frontal	26	29.2
Occiput	51	57.3
Nil	12	13.5

Figure 1: Prevalence of Post Dural Puncture Headache

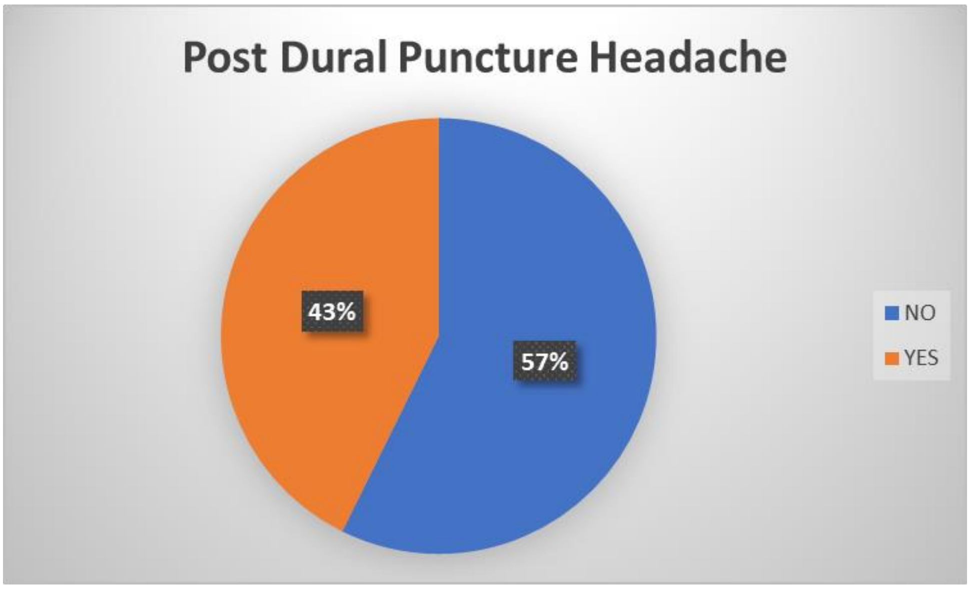

Associated Symptoms

In this study, the most associated symptom was Neck Pain 14 (15.7%) followed by a combination of Back Pain and Neck Pain 9 (10.1%).

Figure 2: Associated Symptoms of Post Dural Puncture Headache

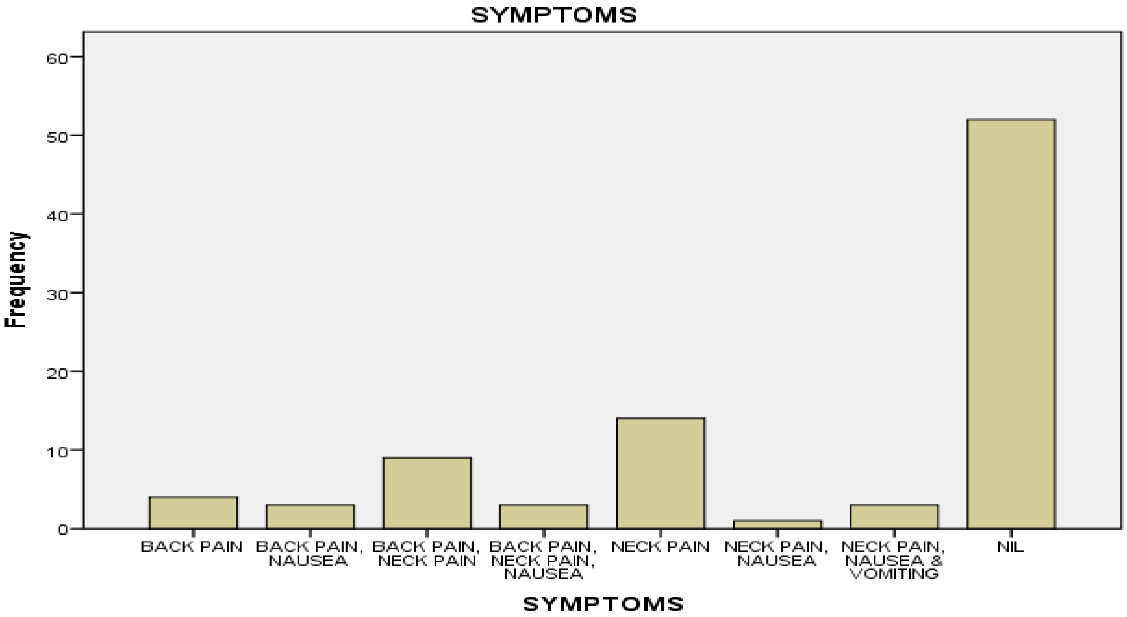

Association Analysis

Post Dural Puncture Headache and Socio-demographic characteristics

There was no statistically significant association with *p-value* ($p \leq 0.05$) between Post Dural Puncture Headache and the socio-demographic characteristics, except Level of Education with *p-value* of ($p=0.035$) and *Cramer's V* value of ($v=0.341$). Which means a weak association between PDPH and Level of Education.

Table 6. Relationship between Post Dural Puncture Headache and pregnant women Socio-demographic characteristics. *P<0.05.*

Socio-demographic characteristics	*Post Dural Puncture Headache*	
	P-Value	***V***
**Age**	*0.25*	*0.21*
**Address**	*0.41*	*0.49*
**Marital Status**	*0.38*	*0.09*
**Religion**	*0.82*	*0.03*
**Level of Education**	*0.035*	*0.34*

Post Dural Puncture Headache and Intraoperative clinical characteristics

There is a weak statistically significant association between PDPH and Gestational age with *p-value (p=0.02)* and *Cramer's V (V=0.29)* and a weak statistically significant association between PDPH and number of attempts with *p-value (p=0.01)* and *Cramer's V (V=0.27).* There are weak statistically significant associations between PDPH and Level of Puncture and Number of CSF drops, with p-values (p=0.01) and (p=0.01) respectively. The Cramer's V are (V=0.44) and (V=0.57) respectively. There are no significant associations between PDPH and the rest of the clinical characteristics.

Table 7: Relationship between Post Dural Puncture Headache and pregnant women clinical characteristics. ***P<0.05.***

**Intraoperative clinical characteristics**	***Post Dural Puncture Headache***	
	P-value	***V***
Parity	*0.20*	*0.14*
Gestational Age	*0.02*	*0.29*
Urgency of CS	*0.15*	*0.15*
Number of attempts	*0.01*	*0.27*
Level of Puncture	*0.48*	*0.13*
Needle Size	*0.01*	*0.44*
Dosage	*0.69*	*0.04*
Number of CSF drops	*0.01*	*0.57*
Previous history of PDPH	*0.17*	*0.15*

Discussion

Regarding prevalence of PDPH there are many studies in this field more so now that spinal anaesthesia has become a predominant anaesthetic procedure for caesarean section in our sub-region. In this study the prevalence of PDPH is 42.7%. Some prospective cohort studies conducted in rural India and Northern Nigeria reported 11.4% and 15.8% incidence of PDPH respectively (5, 6). However, a retrospective cohort study conducted in Jordan reported a lower incidence of PDPH (6.3%) (7).

A cross sectional study conducted in Ethiopia had incidence of post dural puncture headache 31.3% and (32.5%) in Cairo University, Egypt (8, 13). These studies were all prospective just like our study, but the population size and the gauge of the needle differs. In this ~~our~~ study the majority (68.5%) had 22G gauge spinal needle which was associated with PDPH with p-value (p=0.01). This is similar to what is reported by other scholars working elsewhere (14, 15, 16). However, the smaller the needle size the lower the incidence of PDPH. This was demonstrated by the study conducted in India (5) and Nigeria (6) that used 26G and 25G needles respectively. The sample size also influenced the prevalence reported by different scholars working elsewhere. Our study had small sample size compared to other published reports (17, 18) and this may contribute to high prevalence of PDPH we reported.

A retrospective cohort study conducted in Jordan, had a lower incidence of PDPH (6.3%) and repeated attempts increased the risk of its occurrence (7). The lower incidence could be because it was retrospective and the population size of 630 respondents may have contributed to the lower incidence. Similarly, our study had a slight significant association of PDPH and number of attempts with p-value (p=0.01). The number of attempts may increase the probability of piercing the dura matter repeatedly, which will increase the volume of CSF leak, thus increasing the probability of development of intracranial hypotension & PDPH. This was a similar findings with other studies (19, 20, 21) that repeated attempts may increase the risk of developing PDPH. The proportion of repeated attempts of spinal needles related PDPH reports from a population-based study in University of Basel, Switzerland (4.2%) (22) was lower than our prevalence. However, some other studies did not report significant association between number of attempts and PDPH (23, 24).

The majority (79.8%) of the caesarean sections (CS) were due to emergency obstetrics conditions which may also have majority of the PDPH. The study also revealed that among 89

patients that participated, 18 (20.2%) had elective CS, of which 5 (13.2%) patients developed PDPH, and that 71(79.8%) of patients underwent emergency CS, of which 33 (86.8%) developed PDPH. The severity of PDPH reported by patients in this study was higher when compared with other studies conducted in the sub-region. The study conducted in the Northern part of Nigeria (6) and in Ghana (25), most of them had moderate and mild headaches. However, in this study it was observed that the duration of headaches and its severities last for few hours which was not comparable with other studies conducted elsewhere as the headaches last for days (1 to 5 days) in a study conducted in rural India (5).

A recent study shows the incidence of PDPH decreases with higher gauge Quincke needles as follows: 16 to 19 G, about 70%; 20 to 22 G, 20 to 40%; and 24 to 27 G, 5 to 12% (26). In our study it was predominantly 22G (68.5%) which has a significant high incidence of PDPH which may explain the high incidence of PDPH we reported. Some studies conducted in Pakistan (27, 28) had comparable lower incidence of PDPH because of smaller size spinal needle they used. However, a prospective study conducted in Czech with the use of atraumatic Whitacre and Atre Ucan needles, the incidence of PDPH was remarkably reduced (29).

In this study majority of patients experienced severe pain (19.1%) followed by moderate pain (15.7%), unlike other studies (30, 31). The possible reason could be that this study was done among caesarean section patients which means all were females, that with low threshold for pain (32).

In our study, the Level of lumbar puncture was mostly done at L3-L4 (84.3%), which had no statistical significant association with PDPH, with p-value (p=0.48). approximately seventeen percent (16.9%) of the study population had previous history of PDPH. There was no significant association between PDPH and previous history of PDPH with a p-value of (p=0.17).

Conclusion

This study showed the prevalence and risk factors associated with Post-Dural Puncture Headache (PDPH) following caesarean section. The findings suggests that PDPH is a recognized complication, with a prevalence of approximately 42.7%. the higher the gestation

age and multiple number of attempts of spinal anaesthesia had weak associations with PDPH. The use of larger needle gauges and higher number of CSF drops were identified to have moderate statistical significant relationship with PDPH. Patients that underwent emergency CS, have a higher risk of developing Post-Dural Puncture Headache.

Study limitations

This study was not population-based and mainly included women who underwent Caesarean Section at Edward Francis Small Teaching Hospital (EFSTH). As a result, we were not able to apply the findings of the research to the entire Gambian population. Also the study population was small.

Declarations:

Ethics approval and consent to participate:

Ethical approval was requested and secured from Research and Ethics Committee of Edward Francis Small Teaching Hospital. The letter of approval was used to have access to patients and medical files at the Obstetrics and Gynaecology department. Informed consent was obtained from all participants and personal information were confidential.

Consent to publish

Not applicable.

Availability of data and material:

The datasets generated and/or analysed during this study are available and can be shared on reasonable request. The corresponding author can be contacted for the data if required. The dataset have been uploaded.

Acknowledgments:

None.

Funding:

None.

Conflicts of interest:

There is no competing interests between the authors.

Authors' Information

PI is an Obstetrician/Gynaecologist at EFSTH.

References

1. Olawin AM, M Das J. Spinal Anesthesia (2022). In: StatPearls [Internet]. Treasure Island (FL): StatPearls Publishing; 2023 Jan-. Available at https://www.ncbi.nlm.nih.gov/books/NBK537299/ accessed November 2023.

2. Swarup Sri Varaday. (2018). Subarachnoid Spinal Block. Drugs & Diseases > Clinical Procedures accessed online at https://emedicine.medscape.com/clinical_procedures October 2023
3. Nuhu, Si & Embu, Henry & Shambe, Iornum. (2017). Prevalence of Post-Dural puncture headache among caesarean section patients in North Central, Nigeria Journal of Medical Sciences 11.
4. George T. Vaida MD, Sudheer K. Jain MD, in Complications of Urologic Surgery; -Post-Dural Puncture Headache; (Fourth Edition), 2010.
5. Thakur, Sunil; Sharma, Anupriya; Kaushal, Sushruti et al. 14(Suppl 1):p S209-S213, July 2022. Incidence and Risk Factors of "Postdural Puncture Headache" in Women Undergoing Cesarean Delivery under Spinal Anesthesia with 26G Quincke Spinal Needle, Experience of Medical College in Rural Settings in India 2019.
6. Mohammed A D, Ayyuba R, Salisu I, Nagoma A U, Owolabi L F, Ibrahim A. An analysis of postdural puncture headache in obstetric patients: A study from Kano, Nigeria. Trop J Obstet Gynaecol 2017;34:16-20
7. W.N. Khraise, M.Z. Allouh, K.M. El-Radaideh, R.S. Said, A.M. Al-Rusan. Assessment of risk factors for postdural puncture headache in women undergoing caesarean delivery in Jordan: a retrospective analytical study. Local Reg Anesth, 10 (2017), pp. 9-13
8. Yonas Admasu Ferede, Yonas Addisu Nigatu, Abatneh Feleke Agegnehu, Salh Yalew Mustofa. Incidence and associated factors of post dural puncture headache after caesarean section delivery under spinal anesthesia in University of Gondar Comprehensive Specialized Hospital, 2019, cross sectional study
9. Wail N Khraise, Mohammed Z Allouh, Khaled M El-Radaideh, Raed S Said, and Anas M Al-Rusan, 2017 Mar 17, Assessment of risk factors for postdural puncture headache in women undergoing caesarean delivery in Jordan: a retrospective analytical study
10. Ahmed S, Jayawarna C, Jude E. Post lumbar puncture headache: diagnosis and management. Postgrad Med J. 2006;82(973):713–6.
11. Kassa AA, Beyen TK, Denu ZA. Post dural puncture headache (PDPH) and associated factors after spinal anesthesia among patients in university of gondar referral and teaching hospital, gondar, north west Ethiopia J. Anesth. Clin. Res. 2015;6(536):1–6
12. Castrillo A, Tabernero C, García-Olmos LM, Gil C, Gutiérrez R, Zamora MI, Mendoza A, Rodríguez MF, Guerrero P, Rodríguez-Vico JS. Postdural puncture headache: impact of needle type, a randomized trial. Spine J. 2015;15(7):1571–6

13. Hassan Mohamed Ali, Mohamed Yehya Mohamedb, Yahya Mohamed Ahmedb. Postdural puncture headache after spinal anesthesia in cesarean section: Experience in six months in 2736 patients in Kasr El aini teaching hospital – Cairo University. Egyptian Journal of Anaesthesia. 2014;30(4):383–386.
14. Frank RL. Lumbar puncture and post-dural puncture headaches: implications for the emergency physician. J Emerg Med. 2008;35(2):149–157.
15. Amorim JA, Valença MM. Postdural puncture headache is a risk factor for new postdural puncture headache. Cephalalgia. 2008;28(1):5–8.
16. Kuczkowski KM. Post-dural puncture headache in the obstetric patient: an old problem. New solutions. Minerva Anestesiol. 2004;70(12):823–830.
17. R. F. Iteke, D. U. Mulewa , O.K. Mukuku, et al. (2019). Post-spinal Anesthesia Headaches in Resource-limited Settings: Epidemiological and Clinical Characteristics, Determinants and Predictive Score
18. Nafiu OO, Salam RA, Elegbe EO. Postdural puncture headache in obstetric patients: Experience from a West African teaching hospital. Int J Obstet Anaesth 2007;16:4-7.
19. Lybecker H, Møller JT, May O, et al. Incidence and Prediction of Postdural Puncture Headache A Prospective Study of 1021 Spinal Anesthesias. Anesthes Analg. 1990;70(4):389–394.
20. Kuczkowski KM. Post-dural puncture headache in the obstetric patient: an old problem. New solutions. Minerva Anestesiol. 2004;70(12):823–830.
21. F. Tarekegn, S. Eshetie, A. Aregawi, K. Moges, Assessment of the prevalence and associated risk factors of post dural puncture headache (PDPH) after cesarean section delivery under spinal anesthesia, J Anesth Crit Care Open Access, 8 (6) (2017), Article 00330
22. Seeberger MD, Kaufmann M, Staender S, et al. Repeated Dural Punctures Increase the Incidence of Postdural Puncture Headache. Anesth Analg. 1996;82(2):302–305.
23. Bezov D, Ashina S, Lipton R. Post-dural puncture headache: Part II–prevention, management, and prognosis. Headache. 2010;50(9):1482–1498.
24. Jeanjean P, Montpellier D, Carnec J, et al. Headaches after spinal anesthesia: prospective multicenter study of a young adult population. Ann Fr Anesth Reanim. 1996;16(4):350–353.
25. Imarengiaye C, Ekwere I. Postdural puncture headache: a cross-sectional study of incidence and severity in a new obstetric anaesthesia unit. Afr J Med Med Sci. 2006 Mar;35(1):47-51. PMID: 17209327.

26. Abouleish E, Vega S, Blendinger I, Tio TO. Long-term follow-up of epidural blood patch. Anesth Analg 1975;54: 459-63
27. Bano F, Haider S, Aftab S, Sultan ST. Comparison of 25-gauge, Quincke and Whitacre needles for postdural puncture headache in obstetric patients. J-coll physcians surg pak. 2004;14:647-50.
28. Khaleel Ahmad, Muhammad Javed, Maqsood Zahid. Comparison of Frequency of PDPH in Obstetrical Patients after Caesarean Section under Spinal Anaesthesia with 25 G Quincke(cutting) needle 25 G pencil point needle (non-cutting)
29. L'ubuský M, Berta E, Procházka M, Marek O, Kudela M. Development of incidence of post-dural puncture headache in patients undergoing caesarean section in spinal anaesthesia at the Department of Obstetrics and Gynecology in Olomouc during 2003-2004. Cas Lek Cesk 2006;145:204-8
30. S. Aftab, S. Nur-Ul-Haq, A. Ara, J.A. Hassan, Post dural puncture headache: comparison of 26G Quincke with 25G Whitacre needle for elective caesarean section. Pak J Surg, 25 (4) (2009), pp. 257-261
31. J.M. Shaikh, A. Memon, M.A. Memon, M. Khan, Post dural puncture headache after spinal anaesthesia for caesarean section: a comparison of 25 g Quincke, 27 g Quincke and 27 g Whitacre spinal needles, J Ayub Med Coll Abbottabad, 20 (3) (2008), pp. 10-13
32. W. Admasu, A. Hailekiros, Z. Abdissa, Severity and risk factors of post-operative pain in university of gondar hospital, Northeast Ethiopa, J Anesth Clin Res, 7 (10) (2016), p. 675

Click here to access/download

Supporting Information

Admire EFSTH_REC_2023_097_REC approval _August 3rd 2023.pdf
