# Supplementary material for: Prevalence of dural puncture headache after caesarean section at a tertiary hospital in the Gambia
Source: PLoS One. 2025 Sep 19;20(9):e0303410. doi: 10.1371/journal.pone.0303410 (PMC12448977; doi:10.1371/journal.pone.0303410)
Supplement: Attachment — Submitted filename: Response to the PLOS ONE reviewers.docx [file pone.0303410.s004.docx]

Response to the reviewers: Date: 6^th^ December 2024

We sincerely thank all the reviewers of this article. Is our pleasure to provide responses to the comments and try as much as possible to factor all corrections in the reversed version of the main manuscripts.

Reviewer #1: 
comment; I consider it to be of interest and well developed, since it addresses the issue of headache after the use of epidural anesthesia, one of the undesirable effects in such emotional moments as the birth of a new family member.

Response: We thank you for your kind and supportive comment towards the article. Thank you so very much.

Reviewer #2: The manuscript has many major issues:
1- The language is very poor and MAJOR edits needs to be done. I suggest getting help form an expert English editor or a native speaker before trying to re-submit.

Response: thank you dear reviewer will have reviewed the language with the help of an expert in the language.

2- The methodology is unclear. I can't tell if that was truly cross-sectional, retrospective or prospective study.

Response: the study was a cross sectional study. A cross sectional study can be retrospective or prospectively conducted. In our study it was prospectively conducted.

3- The sample size calculation was not reproducible using the same input reported by the authors.

Response: G power 3.1 software inputs was set at the numbers desired for the study and the output was more than the number we recruited because of the duration of the study.

4- The Introduction part has a plagiarism issue with about 60% of the text being not genuine.

Response : that has been reversed thanking you for the observations.

5- I did not understand why are you reporting participants' religion in your data. If this has any thing to do with the outcome, it must be justified

Response: we do not have justification other than a biodata that was included in the data collection tool. It has no relationship with the subject.

Reviewer #3:

Comments 1:  Although this study is not novel, it could add to information in obstetrics practice
ABSTRACT: The authors stated:"lasting for 3hrs (18%)". Since this is a retrospective study, how did the authors document the duration of the post dural headache.

Response: It was a cross sectional study The data collection and observation of study participants was prospectively conducted.

Comment 2: introduction; the authors should beef up the justification for the study.

Response: thank you for the observation we have added more sentences to justify the study.

Comment 3: METHODS
How did the authors ensure uniformity of patient recruitment? how was the sample size calculated? Was one singe type of spinal needle used in all the participants?
What is the ethical approval number?

Response: patients were recruited consecutively as they were admitted in the labour or antenatal wards. The study identification number is issued when decision to undergo caesarean section was reached. The sample size was calculated using a G power 3.1 software.

Reviewer #4: i have added an attachment with comments as a separate file, kindly go through the file
few of them are: keywords to be arranged alphabetically
address the typographical errors below 18 years are considered as minor and you have included young girls of 15 years, how do you justify it?

Response:

Thank you for your comments. The keywords has been arranged accordingly, the typo errors rectified as much as possible and no study participant that was 15 years. The 15 years was a definition of the lower limit of the age range of reproductive age.
