# Supplementary material for: Prevalence of dural puncture headache after caesarean section at a tertiary hospital in the Gambia
Source: PLoS One. 2025 Sep 19;20(9):e0303410. doi: 10.1371/journal.pone.0303410 (PMC12448977; doi:10.1371/journal.pone.0303410)
Supplement: Attachment — Submitted filename: Response to the reviewers 4.docx [file pone.0303410.s005.docx]

Response to the reviewers: Date: 11^th^ March, 2025

We sincerely thank all the reviewers of this article. Is our pleasure to provide responses to the comments and try as much as possible to factor all corrections in the second reversed version of the main manuscripts.

Reviewer #4: 

Reviewer #4: i have added an attachment with comments as a separate file, kindly go through the file
few of them are: keywords to be arranged alphabetically
address the typographical errors below 18 years are considered as minor and you have included young girls of 15 years, how do you justify it?

Response:

Thank you for your comments.

The file has been downloaded.

Response: The keywords has been arranged accordingly

the typo errors rectified as much as possible and no study participant that was 15 years. The 15 years was a definition of the lower limit of the age range of reproductive age.

The rest of the comments and response has been delivered through the file.

Thank you.

Matthew Anyanwu

Corresponding author
